# Neonatal infection in Sub-Saharan Africa: a cross-sectional pilot study on bacterial pathogens and maternal risk factors

**DOI:** 10.3389/fmicb.2023.1171651

**Published:** 2023-04-25

**Authors:** Simone Blumenröder, Damas Wilson, Edgard Ndaboine, Mariam M. Mirambo, Martha F. Mushi, Oliver Bader, Ortrud Zimmermann, Stephen E. Mshana, Uwe Groß

**Affiliations:** ^1^Institute of Medical Microbiology and Virology, University Medical Centre Göttingen, Göttingen, Germany; ^2^Department of Obstetrics and Gynaecology, Weill Bugando School of Medicine, Catholic University of Health and Allied Sciences, Mwanza, Tanzania; ^3^Department of Microbiology and Immunology, Weill Bugando School of Medicine, Catholic University of Health and Allied Sciences, Mwanza, Tanzania

**Keywords:** neonatal infection, gram-negative bacteria, ampicillin, maternal risk factors Sub-Saharan Africa, Tanzania

## Abstract

**Introduction:**

Although child morbidity and mortality could be reduced in Sub-Saharan Africa during the last years both remain high. Since neonatal infections play a major role, we conducted a cross-sectional pilot study in the lake region of Western Tanzania in order to analyze not only the prevalence of neonatal infection with its bacterial etiology including antimicrobial resistance pattern but also to detect potential maternal risk factors.

**Methods:**

We screened 156 women for potential risk factors and examined their neonates for clinical signs of an infection including microbiological verification. All women were interviewed for medical history and their socio-economic background. High-vaginal swabs (HVS) of pregnant women and blood cultures of sick infants were investigated for bacterial pathogens using culture followed by matrix-assisted laser desorption ionization time-of-flight mass spectrometry (MALDI-TOF MS) or polymerase-chain-reaction (PCR)-based assays. Antimicrobial resistances were determined using a disk diffusion test and verified by VITEK 2. Maternal malaria, blood glucose, and hemoglobin levels were determined by rapid tests and helminth infections by stool microscopy.

**Results and discussion:**

Our results showed a prevalence of 22% for neonatal infections. In total, 57% of them had culture-positive bloodstream infections with Gram-negative bacteria being the most prevalent. All these expressed resistance against ampicillin. The prevalence of maternal infection with helminths or *Plasmodium* was low, indicating that anti-worming strategies and intermittent preventive treatment of malaria for pregnant women (IPTp) are effective. The study identified maternal urinary tract infection (UTI) and an elevated blood glucose level as potential maternal risk factors for early neonatal infection, an elevated blood glucose level, and maternal anemia for a late-onset infection.

**Conclusion:**

Our study, therefore, indicates that monitoring maternal UTI in the last trimester as well as levels of maternal hemoglobin and blood glucose might be important to predict and eventually manage neonatal infections. As Gram-negative bacteria with resistance to ampicillin were most prevalent in culture-proven neonatal sepsis, WHO recommendations for calculated antibiosis in the sick young infant should be discussed.

## Introduction

The reduction of infant mortality rate (IMR) was one of the Millennium Development Goals (MDG) set up by the United Nations (UN) in 2000 (World Health Organization, WHO, 2018). Although the infant mortality rate could be decreased from 9 million deaths of infants younger than 1 year in 1990 to 4.6 million in 2005 (Roser et al., 2019), it still fluctuates drastically throughout the globe with high rates in sub-Saharan Africa. In this study, the highest IMR with 68 deaths per 1,000 live births was reported (Alijanzadeh et al., 2016). Children are at the greatest risk for mortality within the neonatal period (Cortese et al., 2016). With 20.3%, the neonatal mortality rate (per 1,000 live births) in Tanzania is still high (WHO, 2021c). Numbers are still far away from the sustainable development goal (SDG) 3.2 set up by the UN to reduce the IMR of under 5-year-old infants to 25 per 1,000 live births and the neonatal mortality rate to 12 per 1,000 live births by 2030. According to the WHO, Tanzania was among the top 10 countries with the highest number of neonatal deaths (43 deaths at 1,000 live births) in 2019 (WHO, 2020). The neonatal mortality rate was high with 21 per 1,000 live births (World Bank, 2019). Referring to the Demographic and Health Survey Tanzania 2015/2016, most newborns (55%) did not receive any postnatal health check (Ministry of Health, 2016). This should urgently be improved as most neonatal deaths occur within the first 48 h of life and therefore contribute to a high mortality rate.

In addition to preterm-birth and intrapartum-related complications, infection remains an important cause of morbidity and mortality within the neonatal period. Commonly known maternal risk factors for neonatal infection in high-income countries are chorioamnionitis, intrapartum maternal temperature above 38°C, delivery below 37 weeks of gestation, membrane rupture beyond 18 h, and vaginal group B streptococcus (GBS) colonization (Herbst and Källén, 2007).

A distinction is often made between early-onset and late-onset sepsis (EOS and LOS) depending on the point in time since the route of infection and the spectrum of pathogens differ. EOS has been variably defined as occurring during the first 72 h after delivery in (preterm) infants hospitalized in the neonatal intensive care unit versus the first week after delivery in term infants (Simonsen et al., 2014). In our study, we followed the German guideline and defined EOS as sepsis within the first 72 h after birth and the onset of LOS at 72 h after birth (AWMF, 2021). Neonatal infection during delivery mainly causes EOS (Polin, 2012). In contrast, LOS mainly results from postnatal infection and is a typical complication of neonates with indwelling devices in intensive care units (ICU) in contrast to EOS, which is associated with maternal obstetrical complications (Nizet and Klein, 2010). As we saw cases in this study where we could not prove a bacterial bloodstream infection, we use the term *neonatal infection* instead of neonatal sepsis and classify it into an early-onset infection (EOI) and late-onset infection (LOI).

Potential risk factors for an early-onset infection (EOI) in low-income countries are HIV, maternal undernutrition, or placental malaria. These risk factors are associated with poor neonatal outcomes in sub-Saharan Africa; however, they have not been investigated in relation to the prevalence of neonatal infection. The purpose of our pilot study was to create a brief overview of various potential and less investigated risk factors such as maternal helminth infection, urinary tract infection, the maternal level of hemoglobin, the blood glucose level, and the socio-economic status. Moreover, we collected high-vaginal swabs (HVS) to evaluate the spectrum of pathogenic microorganisms in the study area and tested them for maternal malaria infection. We determined the prevalence of each risk factor and investigated the impact of a proven or probable neonatal early and late infection.

## Materials and methods

### Study design and patients

This hospital-based, single-center, cross-sectional pilot study was performed during the period of October to December 2015 at the Bugando Medical Center (BMC) and Sekou Toure Regional Hospital (ST) in the city of Mwanza/Tanzania. The Joint Catholic University of Health and Allied Sciences/Bugando Medical Center ethics and review committee approved the study on 30/7/15 (CREC/089/2015). Ethical clearance was granted by the Ethical Committee of the University Medical Center Göttingen (5/7/22). Inclusion criteria were pregnant women and mothers (age 18 to 45 years) of neonates (age less than 28 days) admitted to either BMC or ST or having attended antenatal care (ANC) visits, uncomplicated pregnancies, and informed consent. Inclusion criteria for neonates were age 0–28 days, signs of infection at delivery or within the neonatal period (retrospective arm of the study), and mothers of neonates, who were followed up by phone calls for the duration of the neonatal period (prospective arm of the study, see below). Exclusion criteria were neonates with congenital malformation, chronic diseases, gestational age < 32 weeks, VLBW (less than 1,500 g), and missing clinical information.

There were two study arms. The retrospective arm of the study (cases) consisted of mothers of a sick neonate in the prematurity unit (PREMU) or neonatal intensive care unit (NICU) at BMC who were traced back. The prospective arm of the study (controls) consisted of pregnant women attending medical care services at BMC or ST, who were examined and followed up after delivery for the duration of the neonatal period by phone calls. Thus, we investigated infants admitted to the hospital with suspected infections (cases) and created a control group of supposed healthy infants from the same region who were monitored by telephone interviews.

### Investigations

A high-vaginal swab (HVS) was taken from pregnant women, and they were asked to provide a stool sample. In addition, quick blood tests were performed for the determination of hemoglobin value (HB), random blood glucose (RBG), and malaria (MRDT). Moreover, all women were interviewed using a standardized questionnaire about demographic data, socio-economic data (education, occupation, and residency), living environment, and state of health including medical and obstetric history. The questionnaire and the informed consent form were translated into Kiswahili by local residents of the Department of Obstetrics and Gynecology. Since the socio-economic aspect was only a minor part of the study, we refrained from using a complex model and focused on education as the main pivotal aspect. We defined a low socio-economic status as having no formal education or incomplete primary school of the mother. Newborns were either checked for clinical signs of neonatal sepsis (compare Table 1) on the wards or were monitored *via* calling their mothers three times throughout the neonatal period (days 3, 15, and 28) to differentiate between early (day 3) and late-onset complications (day 15 and day 28). Clinical aspects of all newborns with a suspected infection on the wards, i.e., temperature, respiratory rate, heart rate, and neurological symptoms were documented. As per hospital standards, blood cultures were taken if neonatal sepsis was clinically suspected. Clinical features were documented using a standardized protocol (Klein, 2001).

**Table 1:** Clinical aspects of sick neonates with proven and probable EOI and LOI.

		EOI		LOI	
Variable		Total	*N* (%)	Total	*N* (%)
Temperature (°C) (36–37.5)	<36.0	20	3 (15.0)	14	1 (7.1)
	36–37.5		0 (0.0)		0 (0.0)
	>37.5		17 (85.0)		13 (92.9)
Pulse (bpm) (100–180)	<100	21	2 (9.5)	14	0 (0.0)
	100–180		17 (81.0)		14 (100)
	>180		2 (9.5)		0 (0.0)
Capillary Refill (sec)	0–3	21	19 (90.5)	13	13 (100)
	> 3		2 (9.5)		0 (0.0)
Breathing (per min) (40–60)	<40	21	3 (14.3)	13	0 (0.0)
	40–60		12 (57.1)		11 (84.6)
	>60		6 (28.6)		2 (15.4)
Cyanosis	Yes	12	2 (16.7)	6	1 (16.7)
	No		10 (83.3)		5 (83.3)
Jaundice	Yes	21	4 (19.0)	14	3 (21.4)
	No		17 (81.0)		11 (78.6)
Periumbilical redness	Yes	21	1 (4.8)	13	2 (15.4)
	No		20 (95.2)		11 (84.6)
Vomiting	Yes	21	4 (19.0)	14	1 (7.1)
	No		17 (81.0)		13 (92.9)
Seizures	Yes	21	8 (38.1)	14	0 (0.0)
	No		13 (61.9)		14 (100)
Activity	Hypotonia	21	5 (23.8)	13	2 (15.4)
	Normal		13 (61.9)		11 (84.6)
	Hypertonia		3 (14.3)		0 (0.0)
Grimace	Poor cry	21	4 (19.0)	14	1 (7.1)
	Normal		12 (57.1)		9 (64.3)
	Screaming		5 (23.8)		4 (28.6)
Discharge of urine (within 24 h)	Yes	21	19 (90.5)	13	13 (100)
	No		2 (9.5)		0 (0.0)
Discharge of meconium (within 48 h)	Yes	21	15 (71.4)	13	10 (76.9)
	No		6 (28.6)		3 (23.1)

### Microbiological analysis

High-vaginal swabs (HVS) were collected using Stuart transport media (HiMedia, India). COS Columbia blood agar, enriched with 5% sheep blood (bioMérieux, Lyon, France) and MacConkey (Oxoid, United Kingdom) agar, were used to screen at 37°C for 18–24 h incubation time for common bacterial microorganisms causing neonatal sepsis, e.g., *Escherichia coli, Streptococcus agalactiae (GBS),* or *Listeria monocytogenes.* Basic microbiological investigations, such as microscopy, biochemical identification, and disk diffusion tests according to the European Committee on Antimicrobial Susceptibility Testing (EUCAST) were performed in the laboratory of BMC as per local standards. Blood cultures were analyzed with a commercial blood culturing system (BacT Alert, bioMérieux). They were incubated at 37°C for at least 72 h. Inoculum of bottles indicating bacterial growth within 72 h were cultivated on blood and MacConkey agar followed by biochemical identification and antimicrobial testing as mentioned above. In order to confirm results, randomly selected bacterial isolates (46.7% of isolates from HVS and 90% of isolates from blood cultures) were transferred to Germany to verify identification by matrix-assisted laser desorption ionization time-of-flight mass spectrometry MALDI-TOF MS (Bruker Daltonics, Bremen, Germany) in the laboratory of the Institute of Medical Microbiology and Virology in Göttingen (UMG). In case of inconclusive MALDI-TOF MS results, sequencing of the 16S rDNA locus was performed. For this, respective bacterial isolates were cultivated on blood agar at 37°C for 18–24 h. A 16S rDNA amplicon was produced using a small portion of the colony as the templates and primers 16s_FWD_27F (5’-GGAGTTTGATCCTGGCTCAG-3′) and 16s_REV_1495 (5’-CTACGGCTACCTTGTTACGA-3′) (Sigma-Aldrich, Germany). After initial denaturation for 4 min at 94°C, PCR conditions using a T3 thermocycler (Biometra, Göttingen, Germany) and 35 cycles were as follows: denaturation at 94°C for 30 s, annealing at 54°C for 30 s, and elongation at 72°C for 90 s. Amplified DNA was purified using nucleospin columns (Macherey Nagel, Düren, Germany). Subsequently, Sanger-based DNA sequencing was performed on the amplicon from both ends using each amplification primer (SeqLab, Göttingen, Germany) and the species were identified by searching against the NCBI Genebank (type strains only). Antimicrobial susceptibilities were confirmed by using the VITEK 2 system version 07.01 (bioMérieux, Nürtingen, Germany).

### Detection of helminth infection

Kato-Katz thick smear is a semi-quantitative technique to detect helminthic infections, especially infections caused by *Schistosoma* spp. as well as soil-transmitted helminths (e.g., *Ascaris lumbricoides*).^1^ It is a diagnostic tool recommended by the WHO in regions with high transmission rates (WHO, 2012). The smear has to be systematically examined under a light microscope (Olympus Corporation, Tokyo, Japan, Model CX21FS1) within 30–60 min after proper preparation. Eventually, the number of eggs recorded per gram of feces is multiplied by 24.

### Rapid tests

Rapid measurement systems were implemented in both in- and outpatient situations as a quick quantitative diagnostic tool, e.g., for the level of glucose and hemoglobin (HB) or to detect a *Plasmodium* infection if no other services are provided.

Under sterile conditions, capillary blood was taken from the fingertip of each woman in order to scale the level of blood glucose (One Touch® Select Glucose-Meter) and the level of hemoglobin (Haemocue ® Hb 201+ System) using rapid measurements. A drop of capillary whole blood was spread on a test stripe. The meters calculate the level of blood glucose in mmol/l, respectively, HB in mg/dl and display the result after a few seconds.^2^

Furthermore, a Malaria Antigen Rapid Diagnostic Test (MRDT) (SD Bioline Ag P.f/pan) was performed. The MRDT is a rapid qualitative and differential test. Histidine-rich protein II (HRP-II) antigen of *Plasmodium* species can be detected. The patient’s blood is mixed with a lysing agent on the test stripe. If a parasitic infection with *Plasmodium* spp. is present, a visible band on the stripe next to the control line will occur within 15 min (WHO, 2021b).

### Statistical analysis

All data were entered into Excel sheets and imported to the statistics program STATISTIKA 13.2 (Statsoft GmbH, Hamburg, Germany) for analysis. Frequencies and cross tables were created to get a first statistical overview of all data. Categorial variables were tested using Pearson chi-squared tests. When the sample sizes were small (<5), Fisher’s exact test was used instead of Pearson chi-squared test. Non-parametric rational data were analyzed using Mann–Whitney *U*-tests. A value of p less than 0.05 was considered statistically significant. Al *p*-values were adjusted referring to Bonferroni correction for multiple comparisons. Multivariate logistic regression was not applicable due to complete separation.

## Results

### Study population

A total number of 194 women and their neonates were enrolled in the study. Due to relevant missing information and other criteria for exclusion, 38 women and their neonates were excluded from the study. Consequently, the study population consisted of 156 women and their neonates. All demographic, socio-economic, obstetrics, and medical variables were either captured in a standardized questionnaire or taken from admission books; laboratory records were taken from laboratory books.

The data were collected in two different ways: neonates of retrospective group A (Cases; *N* = 35) had clinical infectious signs and were seen at BMC within the first 4 weeks after delivery. Of those, 21 had an early-onset infection (EOI), and 14 had a late-onset infection (LOI). Neonates of prospective group B (*N* = 121) were considered healthy (controls). Information about the clinical symptoms of group B was collected by phoning their mothers; they were not seen by medical staff. In all 121 cases, the mothers could be reached on day 3 after delivery *via* telephone to rule out clinical infectious symptoms of an EOI. In total, 66 mothers could also be reached at days 15 and 28 after delivery to rule out an LOI.

**Figure 1:**
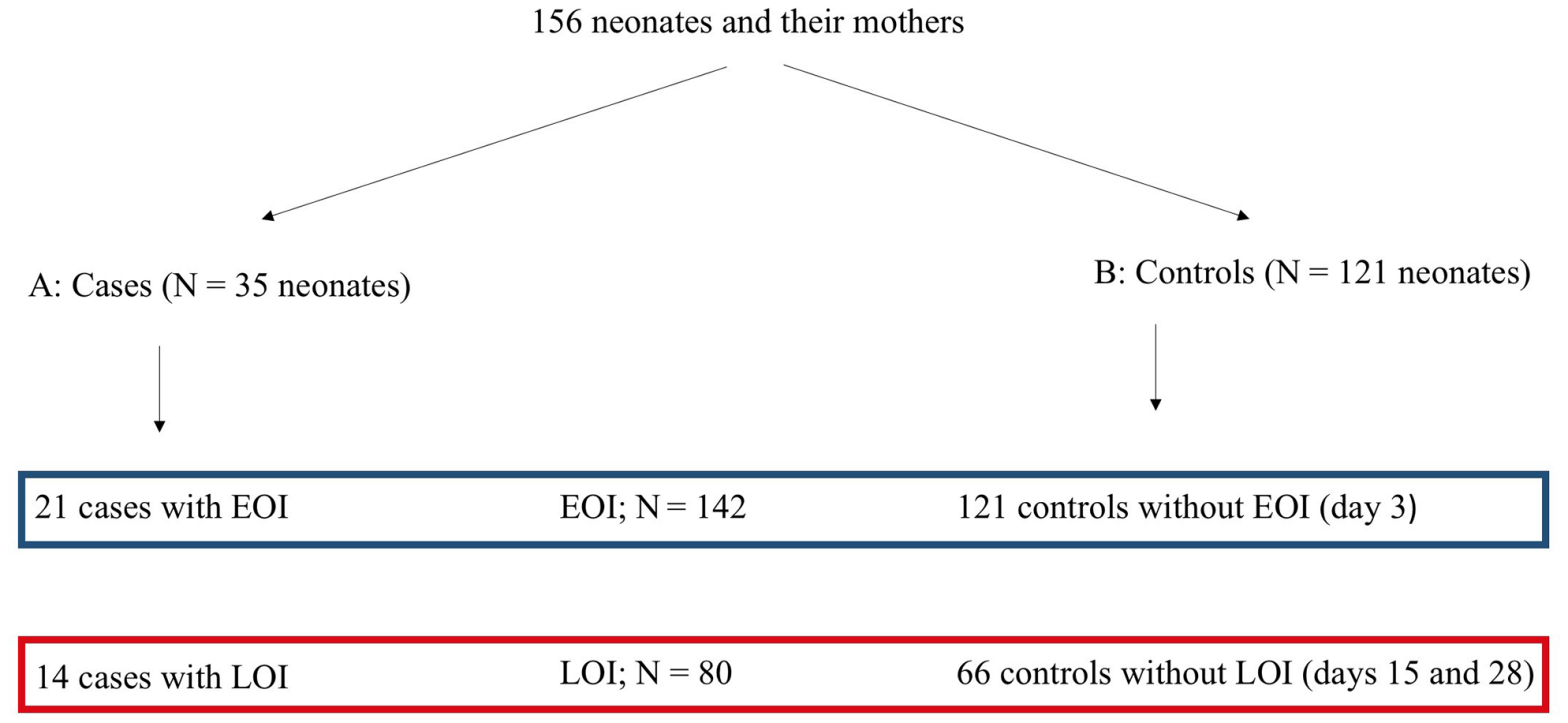
Algorithm of collecting data. NI, neonatal infection; EOI, early-onset infection; LOI, late-onset infection.

We investigated two different but partially overlapping sets of data: the first set included all women and their infants with a proven or clinical EOI (*N* = 21) and the respective controls without an EOI (*N* = 121) with a total of *N* = 142. The second set included all women and their infants with a proven or clinical LOI (*N* = 14) and the respective controls without an LOI (*N* = 66) with a total of *N* = 80. Therefore, the 121 control mothers consisted also of the 66 controls (Figure 1). This division was done to differentiate between maternal risk factors for an early and a late-onset infection.

### Neonatal infection

**Figure 2:**
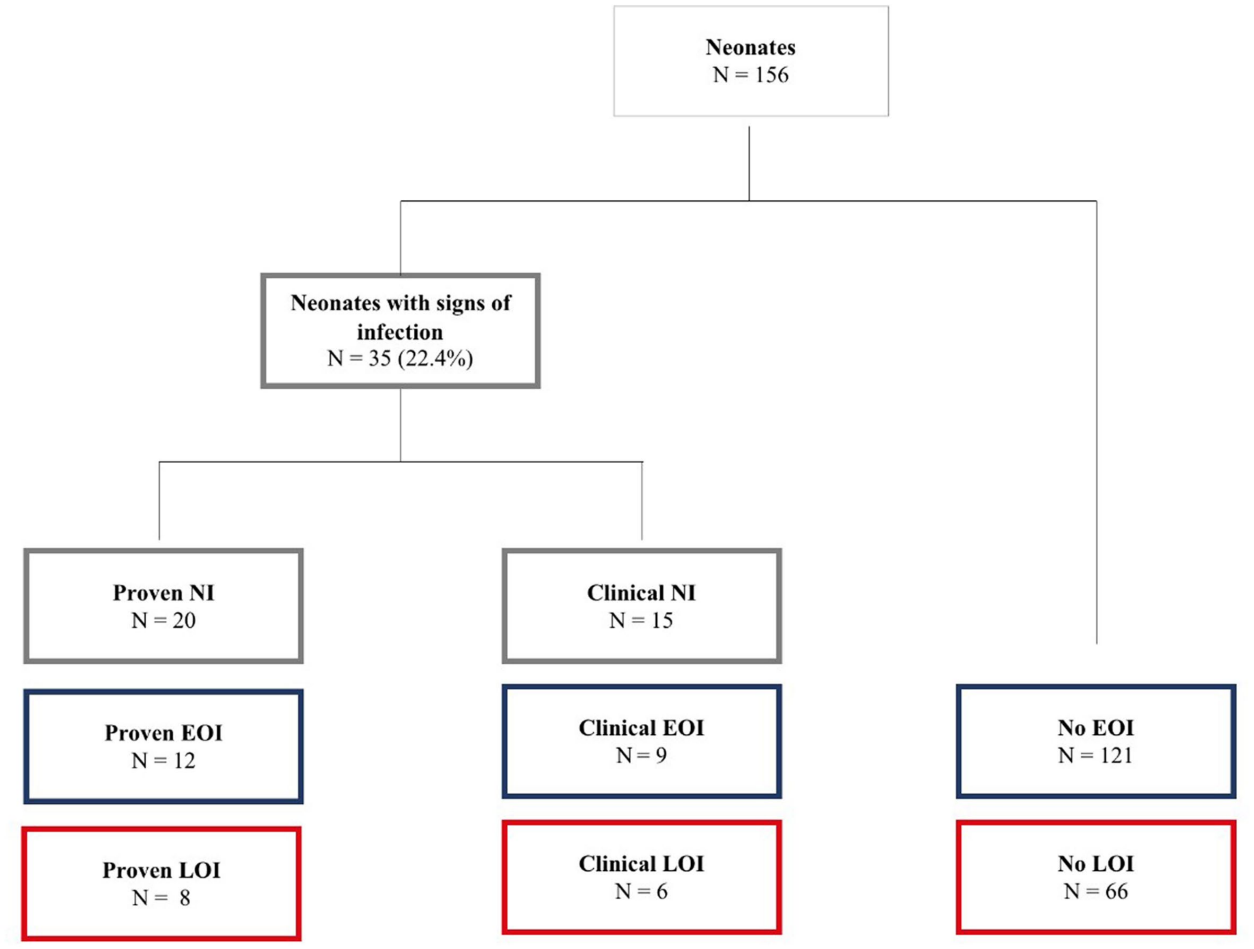
Prevalence of neonatal infection (*N* = 156). NI, neonatal infection; EOI, early-onset infection; LOI, late-onset infection.

When combining the two sets of data, 35 out of 156 neonates (22%) showed clinical signs of infection. Out of these, 20 had a positive blood culture (= proven neonatal infection), indicating EOI in 12 and LOI in 8 children. Neonatal infections without positive blood cultures (= probable neonatal infection) were found in the remaining 15 cases with clinical signs of infection (Figure 2): 10 neonates had clinical signs of infection without bacterial growth in the bloodstream, whereas five neonates showed clinical signs of an infection; however, blood samples were not taken or got lost. In total, 11 neonates had received antibiotics before the blood culture was taken: in five of these cases, blood cultures were positive, and in the remaining six, blood cultures were negative.

The mean birth weight was 3,225 g. No significant differences between infants with and without an EOI were shown for gender and birth weight. The mean gestational age was 38.6 weeks. Neonates who developed signs of an EOI had a lower gestational age (37.3 weeks). Although not significant, infants with EOI were more often delivered by cesarean section than infants without an EOI. Equal gender distribution was found for the infants with and without an LOI. Their mean gestational age was 38.8 weeks. The mean birth weight was 3,229 g. In total, 77% (*N* = 54) of newborns were born by spontaneous vaginal delivery (SVD). No significant differences between infants with and without an LOI were shown for gender, birth weight, gestational age, and mode of delivery.

### Clinical aspects of newborn infants with suspected neonatal infection

All neonates in the prematurity unit (PREMU) and the neonatal intensive care unit (NICU) with proven or probable neonatal infection (NI) were further investigated. A probable neonatal infection was considered as neonates with negative blood cultures but typical clinical symptoms such as variation in temperature, circulatory disturbances, difficulties in breathing, and neurological abnormalities. All infants with an EOI (*N* = 21) and an LOI (*N* = 14) showed at least one symptom indicating a neonatal infection. In total, 17 neonates (81%) with an EOI and 9 (64%) with an LOI presented at least two typical symptoms. Most infants with probable NI had at least two symptoms. Of these 12 (57%) children with an EOI, 10 (71%) were admitted to the hospital because of a fever. Clinical presentations of all sick neonates are shown in Table 1. Bradypnea, convulsions, delayed capillary refill, and hypertonic muscles were only present in neonates with an EOI.

### Microbiological results of bloodstream infections

Overall, with 14 different species, the spectrum of identified bacteria in the blood cultures taken from neonates aged 0–28 days was diverse. Among the 11 Gram-negative bacterial species, the most frequent species were *Salmonella enterica* and *Acinetobacter* spp., followed by *Klebsiella pneumoniae*. Among the Gram-positive ones, the most frequent species were *Staphylococcus haemolyticus, followed by S. aureus* and *Enterococcus* species. We only had a single EOI caused by *Escherichia coli* and no neonatal infection was caused by *Streptococcus agalactiae* or *Listeria monocytogenes* (Table 2). Mixed bacterial growth was found in three blood cultures. In three cases, the bacterial agent could not be identified because their differentiation in Tanzania was finally not conclusive and re-cultivation in Germany failed. One of them had received antibiotics before the blood culture was taken.

**Table 2:** Identified bacterial species in blood cultures using MALDI-TOF MS.

Classification	Clinical isolates	Observations	EOI	LOI
Gram-negative		11	8	3
	*Salmonella enterica*	3 (1/2)*	1	2
	*Acinetobacter* spp.	3 (2/1)*	3	-
	*Klebsiella pneumoniae*	2	1	1
	*Escherichia coli*	1	1	-
	*Pantoea septica*	1 (0/1)*	1	-
	*Pseudomonas aeruginosa*	1	1	-
Gram-positive		10	5	5
	*Staphylococcus haemolyticus*	4	2	2
	*Staphylococcus aureus*	2 (1/1)*	2	-
	*Enterococcus* spp.	2 (0/2)*	1	1
	*Staphylococcus epidermidis*	1	-	1
	*Staphylococcus hominis*	1	-	1
No clear identification	Coliform, CNS, mixed	3	2	1

*(Monomicrobial/polymicrobial).

All bacterial isolates were tested for antimicrobial drug resistance by VITEK 2 system. All *Staphylococcus aureus* isolates were methicillin-susceptible. All Gram-negative bacteria were resistant to ampicillin, which is often recommended as a treatment for sick young infants age up to 2 months referring to WHO recommendations (WHO, 2019). In addition, resistance against third-generation cephalosporins was found in all *Salmonella enterica* and *Acinetobacter* spp. isolates (data not shown).

### Mortality rate

Four neonates (11% of neonates with proven or probable infection) died most likely because of EOI (*N* = 2) or LOI (*N* = 2). Except for one (missing information), they were born on term by spontaneous vaginal delivery with a normal gestational weight. Blood culture was positive only in the two EOI cases (case 1: *S. enterica/E. faecium* unsuccessfully treated with ampicillin, gentamicin, and metronidazole; case 2: *S. haemolyticus, no information about treatment*).

### Maternal risk factors

#### Personal data and medical history

The mean age of all mothers of infants with and without an EOI or LOI was 25.8 or 25.6 years, respectively. Control mothers tended to be younger than mothers of infants with EOI/LOI. We found significant differences in the number of live births (parity) between mothers of infants with an EOI and healthy control neonates (*p* = 0.005, adjusted *p* = 0.05). Significant differences between mothers of infants with an LOI and healthy control neonates were only found before the Bonferroni correction (*p* = 0.007, adjusted *p* = 0.07). All other characteristics were without significance: marital status, gravidity, occupation, residence, low socio-economic status (as defined by no formal education/incomplete primary school), antenatal care visits, and gravidity (data not shown).

According to the medical history, the majority of all women have received a test for HIV and other STDs during the previous pregnancy. However, there were no significant differences between mothers of infants with EOI and the controls for HIV or STDs. Although diabetes was more often reported from mothers of an infant with EOI (11%, *N* = 2 versus 7%, *N* = 7), this difference was not significant. Similarly, no significant differences between mothers of infants with LOI and the controls were found for HIV, STD, and diabetes.

#### Maternal infections

We determined the prevalence of each potential maternal risk factor for infants with an EOI and infants with an LOI, using Pearson’s chi-squared tests or–if sample sizes were small–with Fisher’s exact test: vaginal bacterial colonization, urinary tract infection, helminth infection, *Plasmodium*, and low socio-economic status. Nearly one-third of the study participants showed vaginal bacterial colonization; however, based on Pearson’s chi-square test, we found no statistically significant correlation between women with vaginal bacterial colonization and neonates with a proven or probable neonatal infection (Table 3).

**Table 3:** Prevalence of maternal risk factors for proven/probable EOI and LOI.

EOI; maternal risk factors	Prevalence *N*/tested (%)	EOI cases *N*/tested (%)	EOI controls, *N*/tested (%)	*p*-value	Adjusted *p*-value
Vaginal bacterial colonization*	40/134 (29.9)	8/16 (50.0)	32/118 (27.1)	0.06	0.42
Infection with helminths	8/60 (13.3)	3/10 (30.0)	5/50 (10.0)	0.08	0.56
*Plasmodium* infection	5/135 (3.7)	1/17 (5.9)	4/118 (3.4)	0.5	1
UTI during pregnancy	55/127 (43.3)	11/15 (73.3)	44/112 (39.3)	0.01	0.07
LOI; maternal risk factors	Prevalence *N*/tested (%)	LOI cases *N*/tested (%)	LOI controls, *N*/tested (%)	*p*-value	Adjusted *p*-value
Vaginal bacterial colonization*	23/78 (29.5)	6/13 (46.2)	17/65 (26.2)	0.1	0.7
Infection with helminths	6/36 (16.7)	2/6 (33.3)	4/30 (13.3)	0.2	1
*Plasmodium* infection	4/76 (5.3)	0/13 (0.0)	4/63 (6.3)	1	1
UTI during pregnancy	33/75 (44.0)	6/11 (54.5)	27/64 (42.2)	0.4	1

*Presence of at least one potential bacterial pathogen in high vaginal swabs (HVS).

Among the Gram-negative bacterial species, the most frequent species were *E. coli* (*n* = 25), followed by *Pseudomonas aeruginosa* (*n* = 8), *Acinetobacter* spp. (*n* = 7), *Klebsiella pneumoniae* (*n* = 5), and *Proteus mirabilis* (*n* = 2). In total, six of eight mothers (75%) of an infant with proven or probable EOI were colonized with *E. coli*. In the two other cases, we identified *P. mirabilis* and *Acinetobacter* spp. as potential pathogens. A similar situation was found in mothers of an infant with proven or probable LOI: the vagina of four out of six mothers (67%) was also colonized with *E. coli*; the other two mothers with *K. pneumoniae*.

**Figure 3:**
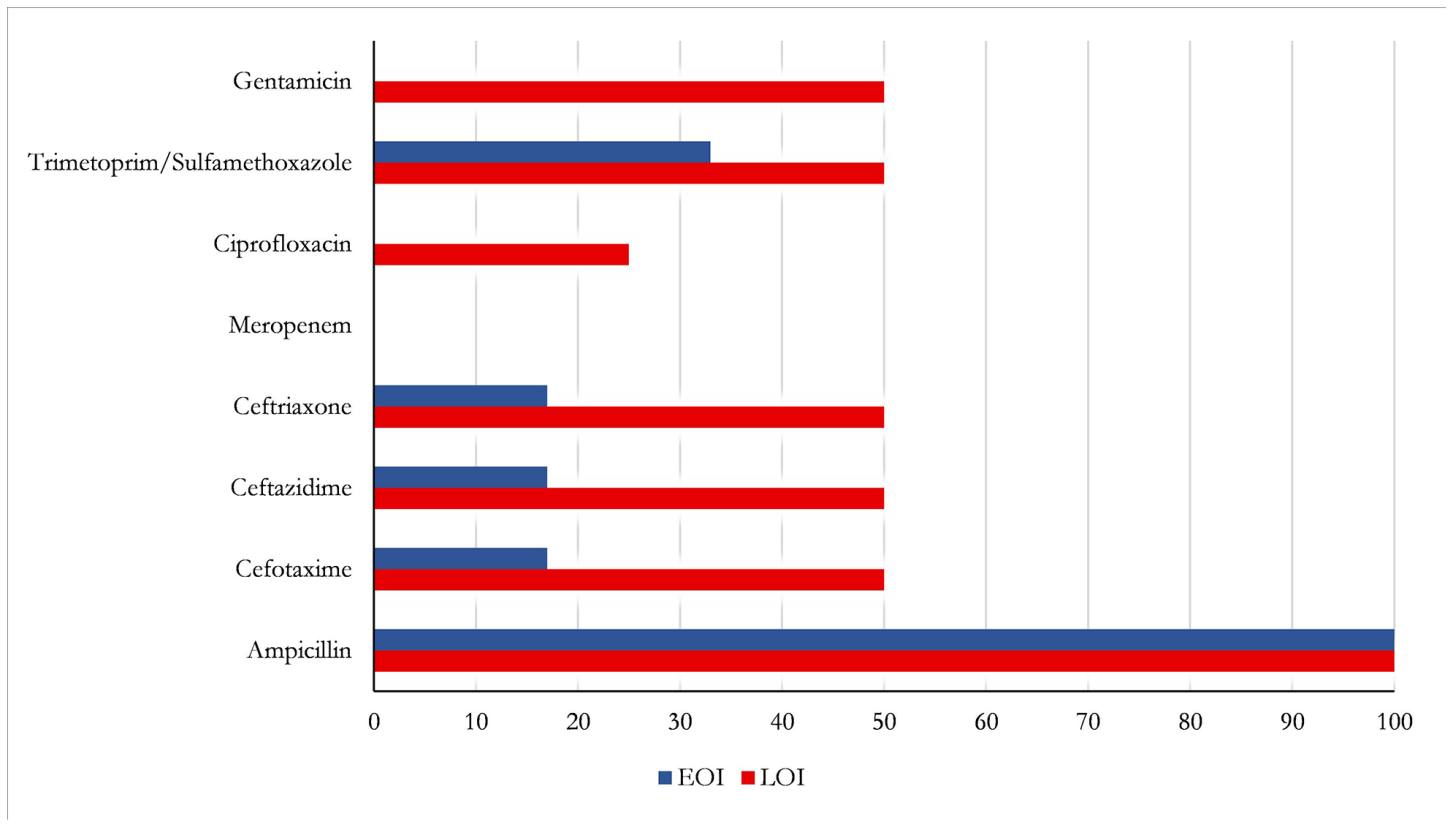
Rates of antibiotic-resistant *E. coli* colonizing the vagina of mothers who gave birth to infants with EOI (*N* = 6; blue) or LOI (*N* = 4; red).

When focusing on the resistance pattern of all *E. coli* strains isolated from the vagina of those pregnant women who gave birth to a newborn with EOI (*N* = 6) or LOI (*N* = 4), the following results were obtained: two were ESBL-producing *E. coli*. Among mothers of infants with EOI, all tested strains (*N* = 5, 1 missing information) were resistant to ampicillin, the antibiotic aside from gentamicin used for the treatment of neonatal infection globally. All *E. coli* strains were sensitive to gentamicin, ciprofloxacin, and meropenem. All *E. coli* strains from mothers of infants with LOI were resistant to ampicillin (*N* = 4), and 50% (*N* = 2) were resistant to all cephalosporines and gentamicin (Figure 3). No antibiotics were given to mothers with bacterial colonization of the vagina because they were asymptomatic and had already delivered their children.

Almost half of all mothers, 43% (*N* = 55) of infants with and without an EOI, and 44% (*N* = 33) of infants with and without an LOI reported to have had a urinary tract infection in the last trimester of pregnancy. Although findings were statistically significant for a proven or probable EOI before Bonferroni correction (73%, *N* = 11), the results do not indicate statistical significance for mothers of infants with a proven or probable LOI (Table 3). Neither maternal infection with helminths nor maternal *Plasmodium* parasitemia showed significant association with a proven or probable EOI or LOI, respectively.

The effects of helminth infection and *Plasmodium* parasitemia were adjusted for potential confounders. For example, we monitored the possible impact of helminth infection by the preventive treatment that was applied during pregnancy according to nationwide recommendations (WHO, 2017). The majority of women received helminthic prevention/therapy (mothers of infants with/without EOI: *N* = 76%, *N* = 105, 4 missing observations; mothers of infants with/without LOI 73%, *N* = 58, 1 missing observation). Hence, we investigated the incidence of helminth infections during pregnancy and compared women who received anthelminthics with those that did not receive deworming medicine. A high percentage of those that received preventive chemotherapy (mebendazole, 500 mg/day) were negative for a helminth infection (mothers of infants with/without EOI: 89%, *N* = 42; mothers of infants with/without LOI: 88%, *N* = 22). Only 11% (*N* = 5) of all mothers of infants with and without an EOI and 12% (*N* = 3) of mothers of infants with and without an LOI had a helminth infection despite chemoprophylaxis. Yet, the findings were not statistically significant.

Most of all women (mothers of infants with/without EOI: 84%, *N* = 116, 4 missing information; mothers of infants with/without LOI: 80%, *N* = 63, 1 missing observation) received intermittent preventive malaria treatment during pregnancy (IPTp) (sulfadoxine-pyrimethamine) according to nationwide recommendations (WHO, 2022). A Pearson’s chi-squared test showed no statistical significance for the association of IPTp and the presence of malaria parasitemia.

#### Maternal hemoglobin and blood glucose levels

To screen for anemia and blood glucose levels, we performed quick blood tests on all women. We defined a hemoglobin (Hb) level below 11 g/dl as anemic according to WHO recommendations (WHO, 2011). The mean hemoglobin level of all mothers of infants with and without a neonatal infection was 11.0 g/dl. Mothers of neonates with proven or probable infection were checked after delivery and were found to have a lower mean level of hemoglobin (9.6 g/dl and 9.8 g/dl for mothers of an infant with EOI and LOI, respectively) compared to those that were checked during a late point of pregnancy (11.2 g/dl and 11.3 g/dl, respectively). Out of 138 women, 124 (89.9%) mothers of infants with and without an EOI reported to have received hematinic supplementation (iron and folic acid) during pregnancy. In total, 83.5% (*N* = 66) of mothers of infants with and without an LOI received hematinic supplementation during pregnancy. Based on a Man–Whitney *U*-test, we found statistical significance for a low level of hemoglobin in mothers and the incidence of neonatal infection (Table 4).

**Table 4:** Maternal level of hemoglobin (g/dl) of mothers of infants with neonatal infection.

	N	Mean	Med	Min	Max	Standard deviation	*p*-value	Adjusted *p*-value
Mothers of infants with EOI	11	9.6	9.5	5.3	13.4	2.4	0.02	0.14
Mothers of infants without EOI	95	11.2	11.2	5.1	17.9	1.8		
Total observations EOI	106	11.0	11.2	5.1	17.9	1.9		
Mothers of infants with LOI	8	9.8	10.3	7.5	10.6	1.1	0.004	0.03
Mothers of infants without LOI	44	11.3	11.5	5.1	17.9	1.8		
Total observations LOI	52	11.0	11.3	5.1	17.9	1.8		

N, number; Med, median; Min, minimum; Max, maximum.

Referring to the WHO, we considered random capillary glucose >11.1 mmol/l as the cut-off value (WHO, 2013). The mean level of blood glucose of all mothers of infants with and without an EOI or LOI was 5.3 mmol/l and 5.4 mmol/l, respectively. Mothers of neonates with EOI had a mean level of random blood glucose of 5.9 mmol/l at the time after delivery. Control mothers of healthy neonates had a mean level of 5.2 mmol/l during pregnancy. The maximum blood glucose level of mothers of neonates EOI was 8.2 mmol/l compared to 12.4 mmol/l in control mothers (Table 5). Mothers of infants with LOI had a higher mean level (6.2 mmol/l) compared to control mothers with healthy neonates (5.3 mmol/l). The maximum blood glucose level in mothers of infants with LOI was lower compared to mothers of infants without LOI (7.2 mmol/l versus 12.4 mmol/l; Table 5). The *p*-value showed a significant correlation between a maternal elevated level of blood glucose and the incidence of neonatal infection (Table 5).

**Table 5:** Maternal blood glucose levels [mmol(l)] of mothers of infants with neonatal infection.

	*N*	Mean	Med	Min	Max	Standard deviation	*p*-value	Adjusted *p*-value
Mothers of infants with EOI	18	5.9	5.8	3.9	8.2	1.2	0.005	0.04
Mothers of infants without EOI	109	5.2	5.1	3.6	12.4	1.2		
Total observations EOI	127	5.3	5.1	3.6	12.4	1.2		
Mothers of infants with LOI	13	6.2	6.4	4.8	7.2	0.8	0.0008	0.006
Mothers of infants without LOI	55	5.3	4.9	3.8	12.4	1.4		
Total observations LOI	68	5.4	5.2	3.8	12.4	1.4		

*N*, number; Med, median; Min, minimum; Max, maximum.

## Discussion

The prevalence rate of neonatal infection in our study was 22% with an estimated incidence of 4%. Incidence rates of neonatal infections in low-income countries are reported to be 6.5–23 per 1,000 live births (reviewed by Vergnano et al., 2005) and 6–21 per 1,000 live births in sub-Saharan Africa as reviewed by Stoll (2001). Since most of these studies were hospital-based, numbers most likely are underestimated because EOI of infants born at home or LOI with manifestations after discharge from the hospital might be missed.

Among all neonates with clinically suspected infections, we determined a prevalence rate of 57% for culture-proven sepsis. Two other studies from BMC presented conflicting results: Kayange et al. (2010) determined a rate of 47% for blood culture-positive neonatal infections. Coagulase-negative staphylococci (CoNS) were considered pathogens but repetition of blood cultures in case of potential contamination was not reported. In contrast, Chacha et al. (2014) reported a rate of 20% for blood culture-proven neonatal infections. In this study, CoNS were considered as pathogens when they could be re-isolated. However, in both studies, only cases of EOI were included. The high variability of results for neonatal infection in low-income countries has also been reviewed by Zaidi et al. (2009) who described a positive blood culture rate between 15 and 62% in infants younger than 60 days. Reasons for the wide range can be different clinical diagnosis criteria, different laboratory methods, and different handling in case of unclear identification or suspected contamination. A repeated blood culture can reduce positive rates if results were not confirmed on the other hand counting potential contaminants as pathogens can increase numbers. The high prevalence of seven CNS in our study could be explained, e.g., by including contaminants as potential pathogens without repeating those blood cultures in respective patients.

Unfortunately, there is no clear internationally standardized definition of EOI (Wynn, 2016). Only a few studies agree with the German guidelines (Kayange et al., 2010; Chacha et al., 2014; Akindolire et al., 2016), whereas others extend the definition of EOI to less than 7 days after delivery (Seale et al., 2009; Zaidi et al., 2009; Kohli-Kochhar et al., 2011). Sometimes, the onset of infection might even not be clear as symptoms of neonatal infection are very unspecific. Particularly, in low-income countries, infants are often born outside of health facilities (Mrisho et al., 2007), making a clear differentiation between EOI and LOI even more difficult.

Although the sample size was small, our results on bacterial species identified in neonates are in good agreement with other investigations. A review by Williams et al. (2018) showed that among neonates, Gram-negative bacteria were the predominant cause of EOI. Thaver and Zaidi (2009) also reported non-typhoid *Salmonella* spp. to be the most frequent Gram-negative agents of EOI in low-resource countries. However, especially prevalence rates of neonatal GBS infections vary from 0% (Seale et al., 2009; Mkony et al., 2014; Akindolire et al., 2016) to a higher rate of 17% in Malawi (Milledge et al., 2005). As we could not detect maternal GBS carriers in our study, the lack of GBS as the cause of neonatal infection was not surprising.

Antibiotic resistance is globally increasing (WHO, 2021a). Our result that all Gram-negative bacteria isolated from blood cultures of neonates were resistant to ampicillin is alarming. Data from two different studies from Muhimbili National Hospital in Dar es Salaam revealed similar findings: Mhada et al. (2012) performed a cross-sectional study with 330 neonates with clinical infection for 4 months and reported high resistance rates among all isolates to ampicillin (88%) and to gentamicin (59%). Mkony et al. (2014) presented a resistance rate of 92% to ampicillin and 42% to gentamicin after performing a cross-sectional study on 208 neonates with clinical infection. Based on these data and in light of WHO recommendations (WHO, 2019), first-line calculated treatment with ampicillin should be discussed. Inappropriate usage of antibiotics might become a bigger challenge as antibiotic availabilities in resource-poor settings are limited and there are only a few alternatives. Carbapenems that were highly sensitive in our study are not recommended for infants younger than 3 months (meropenem) or even 1 year (imipenem).

### Maternal risk factors

As expected, statistical analysis showed no correlation between maternal vaginal bacterial colonization and the incidence of EOI or LOI. However, maternal vaginal bacterial colonization would have rather influenced EOI than LOI. As we performed HVS not only in pregnant women but also in post-delivery women, it remains unclear whether the pattern of pathogens has changed after delivery. This might have influenced our findings.

In our study, *E. coli* was the most frequently identified bacterial species colonizing the vagina of pregnant women. All of these isolates were resistant to ampicillin. In one case, ampicillin-resistant *E. coli* were found in both the vaginal swab of the mother and the blood culture of her child. We detected five ESBL-producing *E. coli* isolates. Two of the women with ESBL-producing *E. coli* colonization gave birth to a child with probable neonatal infection (one EOI and one LOI). We found two isolates among all Enterobacterales with a pattern of multidrug resistances (multi-resistant Gram-negatives). It is difficult to find comparative literature as most studies concentrate either on vaginal GBS colonization only or on bacterial vaginosis. Mulu et al. (2015) found a rate of 13% among pregnant women in Ethiopia that have had a bacterial infection including *E. coli*, *Pseudomonas* spp., and *candidiasis*. None of the pregnant women was found to be colonized by GBS. Indeed, the prevalence rates of maternal GBS colonization throughout countries of sub-Saharan Africa seem to differ: Gizachew et al. (2019) presented a recto-vaginal carriage rate from 3.0 to 29% throughout East African countries after reviewing studies from 1989 to 2019. There might be different reasons why we were unable to isolate any GBS in our study. Since we collected HVS and no recto-vaginal swabs, we might have missed some GBS. Furthermore, we did not use a specific culture medium with a higher sensitivity for GBS detection. Similar reasons might apply to the lack of finding *Listeria monocytogenes* in our study population. However, for Europe, the rate of vaginal colonization with *L. monocytogenes* has been described to be either 0% (Lamont and Postlethwaite, 1986) or very low (0.2%; Stepanović et al., 2007). Data for listeriosis in pregnancy in Sub-Saharan Africa hardly exist.

Antimicrobial resistance is increasingly becoming a global health challenge. Our findings on high resistance rates among Gram-negative bacteria especially against ampicillin and ESBL-producing *E. coli* are supported by other studies from Africa: When analyzing 350 pregnant women in Mwanza/Tanzania, Kamgobe et al. (2020) found *E. coli* nearly three times more often in women with premature rupture of the membranes (PROM) (74%) than without PROM (27%). In addition, a high resistance rate to ampicillin was observed. Similarly, Mulu et al. (2015) found more than 80% of all Gram-negative vaginal isolates of women of reproductive age to be resistant to ampicillin. A review by Bulabula et al. (2017) described a prevalence rate of 17% of colonization with ESBL-producing *Enterobacteriaceae* among African pregnant and post-partum women. Using a less sensitive method for susceptibility testing, a lower rate of ampicillin resistance of *E. coli* was found in pregnant women from Mozambique and Morocco (Sáez-López et al., 2016).

Based on medical history, nearly half of all women in our study reported to have had a UTI during pregnancy, indicating a scarce significant correlation between a maternal UTI and the incidence of an EOI. Using actual laboratory diagnosis, another study conducted at BMC and ST in Mwanza presented a prevalence rate of 28% UTI among pregnant women in their second or third trimester (Kaduma et al., 2019). Also at BMC, Masinde et al. (2009) reported a prevalence rate of 32% of symptomatic UTI in pregnant women, although only 18% of them presented with significant bacteriuria. A study from Ghana found also a significant association between a history of UTI during pregnancy and neonatal infection (Siakwa et al., 2014).

We identified only eight pregnant women, who had a helminth infection despite preventive chemotherapy with mebendazole. There was no association between helminth infection during pregnancy and neonatal infection. However, Tanzania is one of several countries where preventive deworming chemoprophylaxis with albendazole or mebendazole after the first trimester is recommended (WHO, 2002). In contrast, in Kenya, a prevalence rate of 32% for schistosomiasis and 33% for hookworm infection was described in pregnant women (Malhotra et al., 2015). Similarly, the prevalence of *Plasmodium* parasitemia was far lower in our study compared to other studies from sub-Saharan Africa (Van Eijk et al., 2008, 2009; Kitron et al., 2013; Malhorta et al., 2015). A reason for this discrepancy could be that the peak of infection throughout pregnancy is in the second trimester (Takem and D’Alessandro, 2013); women we tested were in their last trimester or even post-partum. In addition, we used a rapid diagnostic test with a lower sensitivity than microscopy (Takem and D’Alessandro, 2013). Similar to our result, Willilo et al. (2016) found a low malaria prevalence rate of 13% among pregnant women in Western Tanzania. An explanation for the low prevalence in our study population was the fact that nearly 80% of all women had received anti-malaria medication as is recommended in Tanzania (Ministry of Health, 2016). IPTp seems to be very effective and has been shown to reduce *Plasmodium* infection by 28 to 47% (Takem and D’Alessandro, 2013). We identified significant differences in hemoglobin levels only between mothers of infants with LOI and their controls. A prospective study from the Republic of Benin with a prevalence rate of 40% for anemia in 617 pregnant women confirms our results (Koura et al., 2012). Ouma et al. (2007) indicated malaria as an important risk factor for anemia. We know that the prevalence of HIV, malaria, and helminth infection that can aggravate anemia was low among our study participants. Other reasons for the rather low prevalence of anemia in our study members may be that the majority of them had received folic acid and iron, anthelmintic prophylaxis, and IPTp. There is evidence that maternal anemia contributes to poor pregnancy outcomes: A three times higher mortality rate of infants born to severely anemic women was presented in another Tanzanian study (Marchant et al., 2004). When comparing blood glucose levels between mothers of infants with neonatal infections and the respective controls, we found significant differences for both, mothers of children with EOI and those with LOI. However, we only know the post-delivery blood glucose levels of mothers with sick neonates, whereas screening is usually recommended between weeks 24 and 28 of pregnancy using an oral glucose tolerance test (ACOG, 2017).

Although a low socio-economic status has been described as a risk factor for neonatal health (Thaver and Zaidi, 2009), there was no significant correlation between socio-economic data (as defined by maternal education) and neonatal infection. Similarly, we were not able to identify a significant correlation between antenatal care visits and neonatal infection. However, we only collected data about the mother and did not implicate information about the father as also suggested by Lampert et al. (2014). In addition, our study focused on hospital deliveries. Thaver and Zaidi (2009) reviewed community-based studies with much more heterogeneous study collectives. A cross-sectional survey in southern Tanzania showed that women with at least primary education are more likely to choose health facilities for childbirth (Mrisho et al., 2007). It is conceivable that families with no formal or incomplete education may have a lower income. Consequently, they may avoid antenatal care visits due to transport spending or have a lack of knowledge that might also contribute to a higher rate of home deliveries. Among other factors, home deliveries have been shown to be predictors of neonatal death (Yeshaneh et al., 2021). Future studies should include monitoring infants born at home by medical staff to make sure that the most vulnerable infants are not underrepresented.

During the time of the study, there was a sudden and unpredictable drop in birth rates at BMC due to increased fees. This made it a lot more difficult to recruit women in labor and was one limitation of our study. There were difficulties in collecting information such as APGAR-score and other problems in previous pregnancies by interviewing mothers. Delivery and labor surveillance was often incomplete by medical staff. Stool samples could not be provided promptly. These reasons contribute to missing information in our study.

## Conclusion

The results of our study indicate that anti-worming strategies and IPTp are effective. Monitoring maternal UTI in the last trimester as well as levels of maternal hemoglobin and blood glucose might be important to predict and eventually manage neonatal infections. As Gram-negative bacteria with resistance to ampicillin were most prevalent in culture-proven neonatal sepsis, WHO recommendations for calculated antibiosis in the sick young infant need to be revised.

## Data availability statement

The original contributions presented in the study are included in the article/supplementary material, further inquiries can be directed to the corresponding author.

## Ethics statement

The studies involving human participants were reviewed and approved by The Joint Catholic University of Health and Allied Sciences/Bugando Medical Center ethics and review committee (CREC/089/2015) and the Ethical Committee of the University Medical Center Göttingen (5/7/22). Written informed consent to participate in this study was provided by the participants’ legal guardian/next of kin.

## Author contributions

UG and SM had the initial idea. SB, DW, EN, MMM, and MFM collected the samples and performed the microbiological analyzes with the support of OB and OZ. SB calculated data for statistics. UG wrote the manuscript with the contributions of all authors. All authors read and approved the final version.

## Conflict of interest

The authors declare that the research was conducted in the absence of any commercial or financial relationships that could be construed as a potential conflict of interest.

## Publisher’s note

All claims expressed in this article are solely those of the authors and do not necessarily represent those of their affiliated organizations, or those of the publisher, the editors and the reviewers. Any product that may be evaluated in this article, or claim that may be made by its manufacturer, is not guaranteed or endorsed by the publisher.

## Footnotes

^1^ microbeonline.com

^2^ onetouchmea.com
